# Effect of Temperature and Relative Humidity on the Reaction Kinetics of an Oxygen Scavenger Based on Gallic Acid

**DOI:** 10.3389/fchem.2018.00587

**Published:** 2018-11-27

**Authors:** Astrid F. Pant, Julia Dorn, Matthias Reinelt

**Affiliations:** ^1^TUM School of Life Sciences Weihenstephan, Chair of Food Packaging Technology, Technical University of Munich, Freising, Germany; ^2^Fraunhofer Institute for Process Engineering and Packaging IVV, Freising, Germany

**Keywords:** active packaging, food packaging, oxygen absorber, kinetic model, polyphenol, 3, 4, 5-trihydroxybenzoic acid

## Abstract

Gallic acid (GA) is a potential oxygen scavenger for food packaging applications. In this study we investigated the effect of temperature and relative humidity (RH) on the reaction kinetics of an oxygen scavenger consisting of GA and sodium carbonate. The reaction was described by a second-order kinetic law and the reaction rate coefficient *k* as well as the scavenger capacity *n* were determined from experimental data using a multiple-run downhill simplex method. Both the rate coefficient and the scavenger capacity increased significantly with higher temperatures. At 21°C it was shown that both the rate coefficient and the scavenger capacity increased significantly with higher RH. However, below 54% RH, there was no detectable reaction. For optimum scavenger performance we therefore recommend GA-based scavengers for packaging of food products with a high water activity stored at room temperature. Prior to application, the packaging materials with GA-based scavengers can be stored at 21°C and 54% RH without losing their scavenger activity. The results of this study provide the basis for the functional design of active packaging systems with GA-based oxygen scavengers.

## 1. Introduction

In food packaging technology, oxygen (O_2_) scavengers are used to protect O_2_ sensitive food products (Vermeiren et al., 2003). O_2_ scavengers are substances that bind O_2_ in a chemical reaction, thereby preventing the oxidation of food components. Depending on the packaging system, O_2_ scavengers can either remove O_2_ from the headspace of the package or improve the O_2_ barrier function of the packaging material. Common applications include sachets containing the scavenger in powder form that are added to a package and also packaging films (monolayer or multilayer) incorporating the scavenger (Singh et al., 2011; Realini and Marcos, 2014). Many commercial O_2_ scavengers are based on iron, sulfite, or oxidizable polymers and they have been in use since the 1990s (Rooney, 2005; Saengerlaub and Mueller, 2017). Besides these established systems, the application of polyphenols as O_2_ scavengers has gained attention due to their availability from renewable resources and their high O_2_ absorption capacity (Wanner, 2010). The O_2_ scavenging effect of polyphenols relies on their autoxidation in alkaline media (Tulyathan et al., 1989; Scoccia et al., 2016).

Gallic acid (3,4,5-trihydroxybenzoic acid, GA) is a simple polyphenol that has been tested as an O_2_ scavenger for packaging applications. Langowski and Wanner (2005) first mentioned GA-based O_2_ scavengers consisting of GA and a base. Application tests with GA-based scavengers integrated either into the adhesive layer or into the coating of packaging films were reported by Goldhan et al. (2007) and Wanner (2010). Concerning the extrusion of packaging films containing GA, Ahn et al. (2016) reported the manufacture of monolayer films based on low density polyethylene. Multilayer films combining layers of biobased linear low density polyethylene and polylactide were produced and characterized by Pant et al. (2017). Using these biobased multilayer films it was shown that the O_2_ absorption by GA-based O_2_ scavengers depends significantly on the relative humidity (RH) and temperature, therefore targeting food products with high water activity. However, to date there is no quantitative description of the reaction kinetics of GA-based O_2_ scavengers and how the kinetics can be influenced by the storage conditions.

In the literature, different approaches for characterizing O_2_ scavengers are mentioned. The intuitive concept of O_2_ absorption capacity is usually applied to indicate the amount of O_2_ that can be absorbed by a defined amount of scavenger. To describe the time-dependent absorption of O_2_, first-order kinetic models have been applied in many cases. This approach often resulted in a good fit of the experimental data (Charles et al., 2003; Galotto et al., 2009). However, it is based on the assumption that one of the reactants, i.e. O_2_ or the scavenging substance, is not limiting for the reaction. This can be the case if there is large excess of either O_2_ or the O_2_ scavenger. In most packaging applications, however, this assumption is not valid, meaning that the kinetic parameters cannot be transferred to packaging systems having different initial O_2_ or scavenger contents. Therefore, first-order kinetic coefficients can merely be used to compare different O_2_ scavengers, but they are meaningless for actual packaging design. To overcome this, some research groups have recently successfully applied second-order kinetic models to scavenger reactions: Dombre et al. (2015) and Di Maio et al. (2017) described the O_2_ absorption of polymer-based scavengers, resulting in reaction coefficients independent of the initial reactant concentrations.

In this study we investigated the influence of temperature and relative humidity on the O_2_ scavenging properties of a GA-based O_2_ scavenger. A second-order kinetic model was developed and the model parameters were obtained by fitting the experimental data. The effects of temperature and relative humidity on the parameters were analyzed. The results provide insight into the properties of GA-based O_2_ scavengers in relation to the main storage parameters for typical packaging applications.

## 2. Materials and methods

### 2.1. Preparation of the oxygen scavenger

Gallic acid (GA) monohydrate powder (99%) was obtained from ABCR, Karlsruhe, Germany. At room temperature GA is yellowish-white crystalline powder (Beyer and Walter, 1991). Water-free sodium carbonate (99.8%, Na_2_CO_3_) was obtained from Th.Geyer, Germany.The GA-based O_2_ scavenger (GA-Sc) was prepared by blending gallic acid monohydrate and sodium carbonate in a mass ratio of 2:1. This ratio of GA and carbonate has successfully been used as an O_2_ scavenger in packaging films in previous studies (Pant et al., 2017).

### 2.2. Oxygen absorption measurement

**Figure 1:**
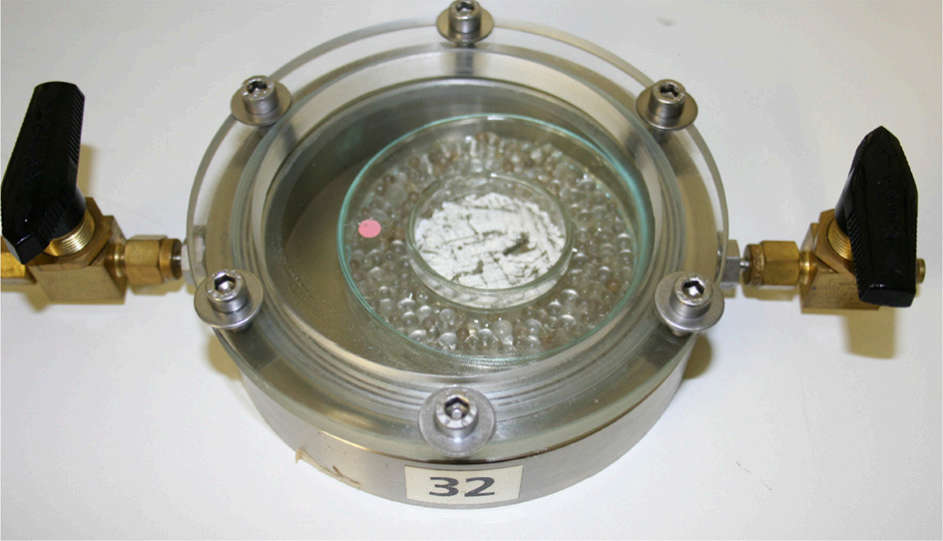
Experimental setup for the oxygen absorption measurements.

For the O_2_ absorption measurements, a thin layer of GA-Sc powder (0.060 g) was spread in a glass bowl and stored in stainless steel cells equipped with two valves for gas flushing (Figure 1). The cells were hermetically closed with a glass lid, sealed with a viton O-ring and had a free headspace volume of 88 cm3 or 108 cm3.

The relative humidity (RH) in the cells was adjusted with silica gel, calcium chloride, sodium chloride, potassium chloride, or water (0% RH, 38% RH, 75% RH, 86% RH, and 100% RH at 21°C, respectively). The storage temperature (5°C, 10°C, 21°C, and 38°C) was adjusted by storing the cells in climatic chambers. The initial headspace gas atmosphere was air. During storage, the decrease in the headspace O_2_ partial pressure (*p*_O_2__) due to the scavenger reaction was monitored non-destructively using Fibox 4 Trace, a luminescence-based oxygen detection system (PreSens Precision Sensing GmbH, Regensburg, Germany). For this, an optical sensor spot (PSt3) was placed inside the cell at the glass top (Figure 1). Absorption data *p*_O_2__ in hPa were transferred to concentrations using the ideal gas law

(1) $$\begin{array}{r} m_{{\text{O}}_{2}}=\left(p_{{\text{O}}_{2}}\cdot V_{\text{HS}}\cdot M_{{\text{O}}_{2}}\right)/\left(R\cdot T\right) \end{array}$$

where *V*_HS_ denotes the headspace volume of the cell, *M*_O_2__ is the molar mass of O_2_, *R* = 8.314 J mol^-1^K^-1^ the ideal gas constant, and *T* is the temperature in Kelvin.

Water vapor partial pressure values p_H2O_ were calculated using the following equations:

(2) $$\begin{array}{r} {\text{p}}_{\text{H}2\text{O}}=\frac{\text{RH}}{100}\cdot e_{\text{sw}} \end{array}$$

(3) $$\begin{array}{r} e_{\text{sw}}=c_{1}\cdot e^{\left(\frac{c_{2}\cdot T}{c_{3}+T}\right)} \end{array}$$

where RH is the relative humidity in %, *e*_sw_ is the saturation vapor pressure over liquid water in hPa, *T* is the temperature in °C and *c*_1_ = 6.112 13 hPa, *c*_2_ = 17.5043 and $c_{3}=241.2^{{}^{\circ}}\text{C}$ are the given model parameters of the Magnus equation (Wernecke, 2003).

For experiments at different temperatures and 100% RH, threefold determinations were made, except for 5°C where only data from two measurements were available. The effect of RH was determined in four parallel experiments for each condition. All results are given as the arithmetic mean together with the standard deviation.

The mean square deviation of the observed O_2_ concentrations was then calculated for each experiment as follows:

(4) $$\begin{array}{r} {\text{MSE}}_{\text{exp}}=\sqrt{\frac{1}{N}\overset{m}{\underset{i=1}{\sum }}\overset{q}{\underset{l=1}{\sum }}{\left({\left[{\text{O}}_{2}\right]}_{\exp}-{\left[{\text{O}}_{2}\right]}_{\text{mean}}\right)}^{2}} \end{array}$$

where *N* is the total number of observations, *m* is the number of parallel experiments, *q* is the number of observations in one experiment, [_O_2_]exp_ is the observed O_2_ concentration and [_O_2_]mean_ is the arithmetic mean of all *m* observations for each *l*.

### 2.3. Mathematical model

The autoxidation of GA under alkaline conditions has been described as a complex multi-step chain reaction leading to different oxidation products (Tulyathan et al., 1989). However, for the application of GA as an O_2_ scavenger in packaging applications, the main focus lies on describing the rates of reactant consumption, i.e., the time-dependent depletion of O_2_ in relation to the used amount of GA. The following overall reaction was assumed:

(5) $$\begin{array}{r} \text{GA}+\text{n}{\text{O}}_{2}\to \text{G}{\text{A}}_{\text{ox}} \end{array}$$

where *n* is a stoichiometric factor and GA_ox_ accounts for the various reaction products. The reaction is assumed to be irreversible.

We approximated the kinetic law of this reaction by the kinetic law of a second-order elementary reaction with the reaction rate *r* being expressed as follows:

(6) $$\begin{array}{r} r=k\cdot \left[GA\right]\cdot \left[O_{2}\right] \end{array}$$

where [GA] and [*O*_2_] denote the concentrations of non-reacted GA and O_2_ in mol m-3, respectively, and *k* is the reaction rate coefficient in m3 mol-1 s-1. Based on this kinetic law, the net rates of consumption of GA and O_2_ were described using the following system of ordinary differential equations (ODE):

(7) $$\begin{array}{rc} \frac{d\left[GA\right]}{dt} & =-k\cdot \left[\text{GA}\right]\cdot \left[{\text{O}}_{2}\right] \end{array}$$

(8) $$\begin{array}{rc} \frac{d\left[{\text{O}}_{2}\right]}{dt} & =-n\cdot k\cdot \left[\text{GA}\right]\cdot \left[{\text{O}}_{2}\right] \end{array}$$

The ODE system gives an initial value problem and was solved in MATLAB R2014a (The MathWorks, Inc., Natick, MA, USA) using the multistep solver ode15s. The model was fitted to the experimental data to obtain the model parameters *k* and *n*. The fit was optimized based on the minimization of the sum of squared residuals (SSQ):

(9) $$\begin{array}{r} \text{SSQ}=\overset{m}{\underset{i=1}{\sum }}\overset{q}{\underset{l=1}{\sum }}{\left({\left[{\text{O}}_{2}\right]}_{\text{sim}}-{\left[{\text{O}}_{2}\right]}_{\exp}\right)}^{2} \end{array}$$

where *m* is the number of parallel experiments, *q* is the number of observations in one experiment, and [_O_2_]sim_ and [_O_2_]exp_ are the predicted and the observed O_2_ concentrations, respectively.

For the minimization of SSQ, the MATLAB function fminsearch was used. This function is based on a Nelder-Mead downhill-simplex algorithm as described by Lagarias et al. (1998), a simple and widely-used algorithm for local optimization. The termination tolerance of the function value was 10^−4^ and the lower bound on the size of a step was 10^−4^ (MATLAB default settings). The optimization terminates when both stopping criteria are fulfilled. Depending on the start values, the downhill simplex algorithm can be stuck in a local minimum and therefore fail to find the optimum combination of *k* and *n*. Therefore the start values were varied systematically in the range of *n* = 0..10 and *k* = 10^−10^..10^10^ so that in total 231 different combinations of *k* and *n* were used. The results were analyzed for an unambiguous total minimum, which was then considered to represent the optimum values for *k* and *n*. As a measure of the goodness of fit, the root mean square error (RMSE) was calculated according to equation 10:

(10) $$\begin{array}{r} \text{RMSE}=\sqrt{\frac{\text{SSQ}}{N-p}} \end{array}$$

where *N* is the total number of experimental observations and *p* the number of fitted parameters.

## 3. Results and discussion

Temperature and relative humidity have been described as the most important factors for the performance of oxygen scavengers (Braga et al., 2010; Polyakov and Miltz, 2010, 2016). In packaging applications, these parameters are determined by the storage environment and/or the packed food product. In the present study, the effect of temperature and relative humidity on the O_2_ absorption kinetics of a novel GA-based scavenger was studied in order to identify possible applications and to provide necessary kinetic information for packaging design.

### 3.1. Effect of temperature on oxygen absorption

**Figure 2:**
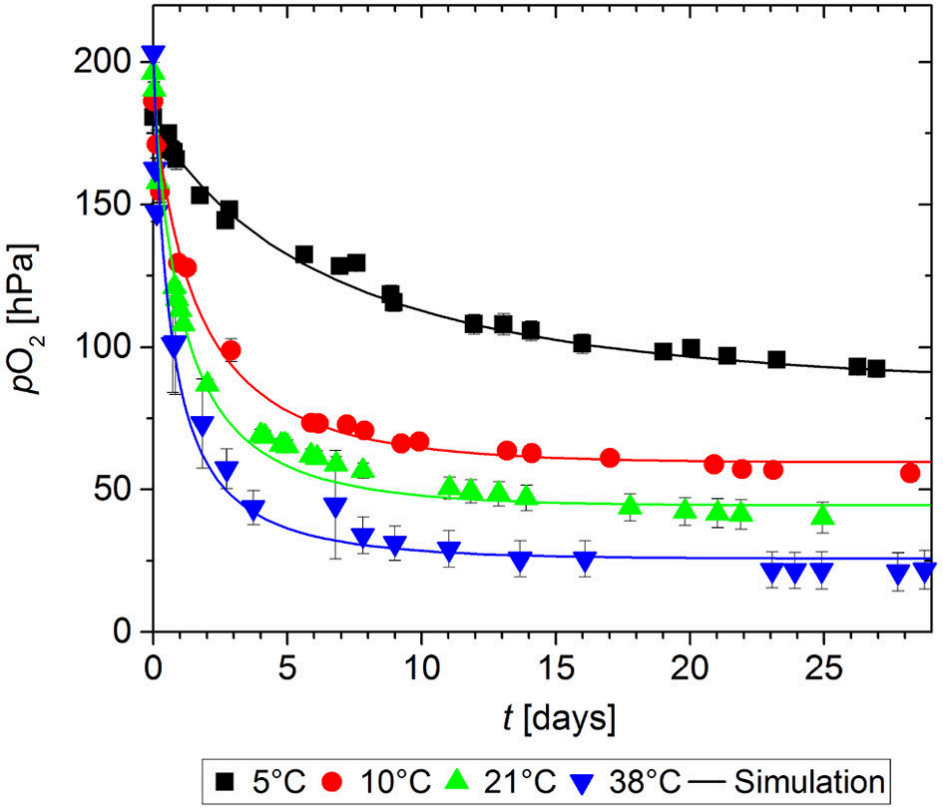
Oxygen absorption by a gallic acid-based oxygen scavenger stored at different temperatures. Symbols show the experimental data and lines show the simulation results.

In order to determine the effect of storage temperature on the O_2_ absorption of GA-Sc, samples of the scavenger were stored at 100% RH at temperatures in the range of 5 to 38°C for 28 days. The chosen temperatures represent typical storage conditions for food products ranging from chilled conditions to tropical conditions. The results of the O_2_ absorption measurements are shown in Figure 2. Higher storage temperatures lead to a faster decrease of the O_2_ partial pressure in the measurement cell. All experimental data sets could be sufficiently described with the chosen kinetic model: The results of the simulation are in good agreement with the experimental values as shown by RMSE values in the order of magnitude of the experimental error (Table 1).

**Table 1:** Model parameters.

**Temperature**	**Relative**	**RMSE**	**MSE_exp_**	***k***	***n***
**^°^C**	**humidity%**	**mol m^−3^**	**mol m^−3^**	**m^3^ mol^−1^ s^−1^**	
5	100	0.135	0.079	2.478·10^−7^	2.03
10	100	0.198	0.090	9.803·10^−7^	2.23
21	100	0.256	0.117	1.496·10^−6^	2.53
38	100	0.469	0.303	2.533·10^−6^	3.49

**Figure 3:**
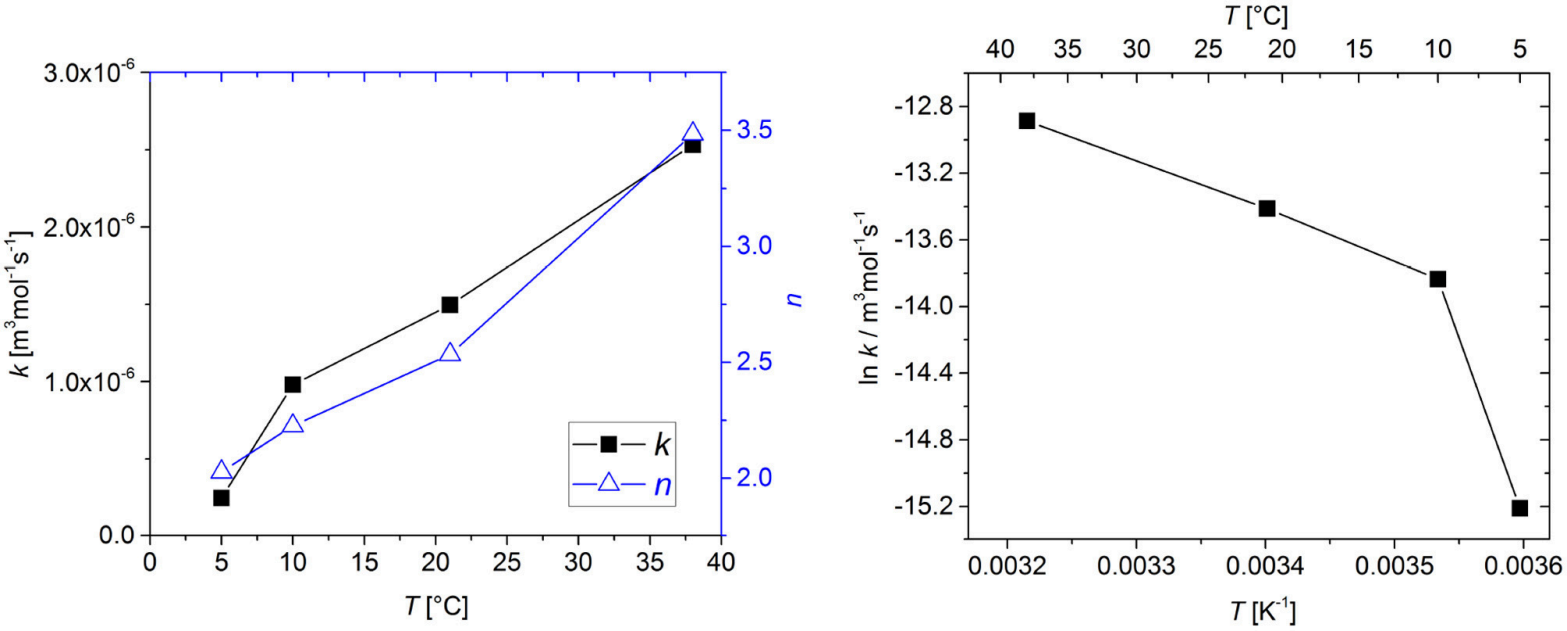
Effect of temperature on the reaction rate coefficient *k* and the stoichiometric coefficient *n* (left) and the associated Arrhenius plot (right). Connecting lines are shown to guide the eye.

The kinetic parameters *k* and *n* were determined by fitting a second-order kinetic model to the available experimental data sets. Table 1 gives an overview of the model parameters. All parameters could be determined using the multiple-step downhill simplex optimization. Figure 3 shows the values for *k* and *n* as a function of temperature.

As could be expected for an oxidation reaction, the rate of O_2_ consumption increased with increasing temperature. However, the effect of temperature cannot be described by the Arrhenius law; only in the 10°C to 38°C range is the Arrhenius plot a straight line. (Figure 3, right). Non-Arrhenius behavior has been observed in many cases where complex reaction chains are described with a simple overall reaction and has also been described for iron-based scavengers (Polyakov and Miltz, 2016).

The stoichiometric coefficient *n* also showed significant temperature dependence. *n* can be interpreted as the scavenger capacity, i.e., the number of O_2_ molecules that are consumed per molecule of GA. The oxidation of GA involves multiple equilibria and parallel or subsequent oxidation reactions and is not yet fully understood (Tulyathan et al., 1989). Both the pathway and the extent of these chain reactions determine the total amount of O_2_ that is consumed and therefore the scavenger capacity. Additionally, the presence of transition metal ions may influence the reaction (Mochizuki et al., 2002). Our results indicate that the reaction pathway and/or the extent of the reaction changes with temperature. However, for a deeper understanding of this temperature dependence more knowledge about the oxidation mechanism is necessary.

### 3.2. Effect of relative humidity on oxygen absorption

**Figure 4:**
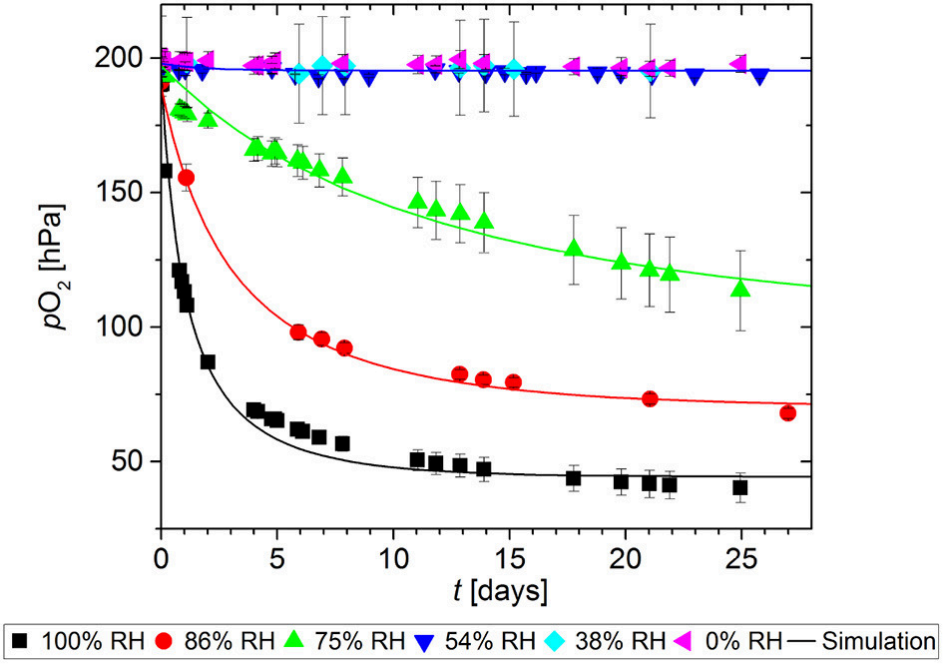
Oxygen absorption by a gallic acid-based scavenger stored at different relative humidities (RH). Symbols show the experimental data and lines show the simulation results.

As with many other oxygen scavengers, the scavenging function of GA-Sc is triggered by humidity (Pant et al., 2017). To quantify the effect of RH on the O_2_ absorption kinetics, samples of GA-Sc were stored at different RH for 27 days. The results are shown in Figure 4. At 0% RH, 38% RH and also 54% RH there was no significant decrease in the O_2_ partial pressure, i.e., no scavenger reaction occurred. In the range from 75% RH to 100% RH there was a faster decrease in the O_2_ partial pressure with higher levels of RH.

The model parameters *k* and *n* were determined by fitting the second-order kinetic model. The results are presented in Table 2. A detailed description of the downhill simplex-based optimization and potential pitfalls are given elsewhere (Pant and Reinelt, 2018).

**Table 2:** Model parameters.

**Temperature**	**Relative humidity**	**RMSE**	**MSE_exp_**	***k***	***n***
**^°^C**	**%**	**mol m^−3^**	**mol m^−3^**	**m^3^ mol^−1^ s^−1^**	
21	0	0.038	0.116	0^*^	0^*^
21	38	0.147	0.179	0^*^	0^*^
21	54	0.051	0.041	0^*^	0^*^
21	75	0.347	0.286	1.348·10^−7^	1.64
21	86	0.136	0.109	5.354·10^−7^	2.01
21	100	0.256	0.117	1.496·10^−6^	2.53

* *no detectable reaction*.

**Figure 5:**
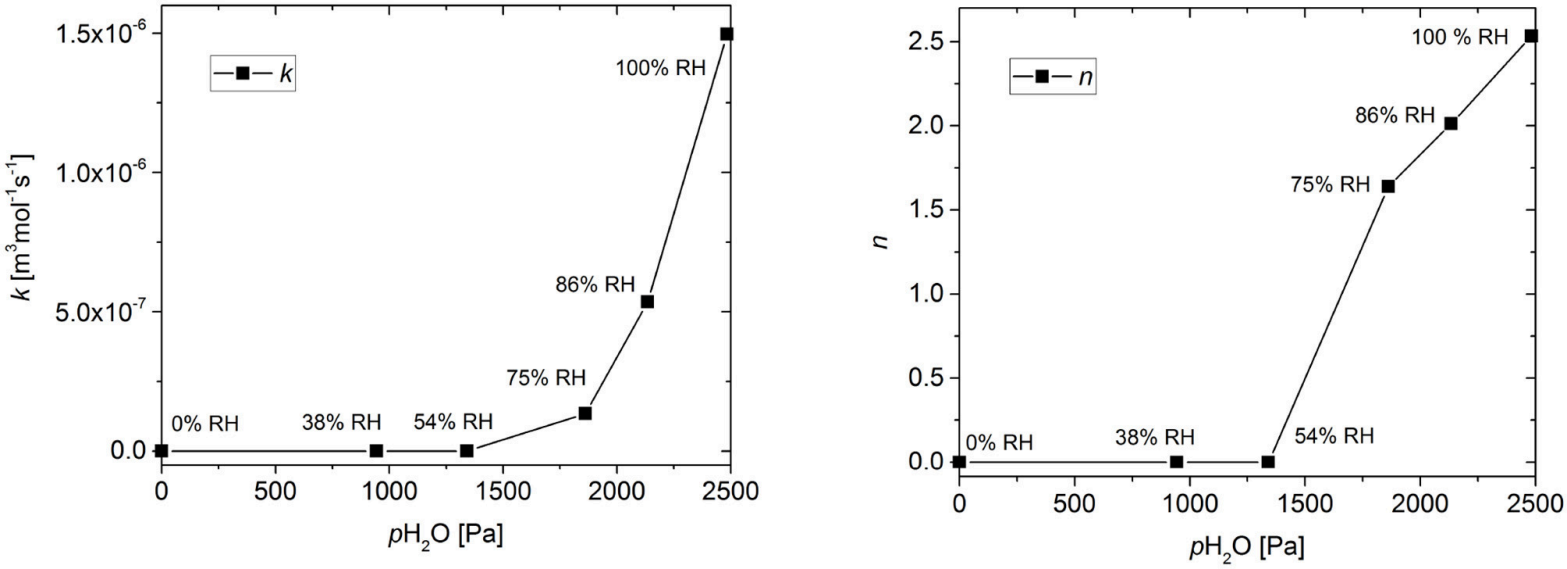
Effect of relative humidity (RH) on the reaction rate coefficient *k* and the stoichiometric coefficient *n*. Connecting lines are shown to guide the eye.

The effect of RH on the model parameters is shown in Figure 5. The values of both the reaction rate constant *k* and the stoichiometric factor *n* show an increase at higher relative humidity, with the threshold being between 54% RH and 75% RH.

These results confirm the triggering effect of humidity on the scavenger reaction of GA. Prior to the reaction with O_2_, the scavenger reaction involves the deprotonation of the GA (Tulyathan et al., 1989; Eslami et al., 2010). Each GA molecule can donate up to four protons. The extent of the deprotonation is determined by the acid-base equilibrium established by the scavenger mixture of GA and sodium carbonate in the presence of water. Here, water serves as the medium for the proton transfer. Therefore, the amount of available water determines if there is a reaction and, in combination with the base, also determines the extend of the deprotonation, which in turn may influence the subsequent oxidation pathway.

## 4. Conclusion

This study investigated the effect of temperature and relative humidity on the scavenger kinetics of a GA-based scavenger. It was shown that both the reaction rate coefficient and the scavenger capacity of the GA-based O_2_ scavenger increased with higher temperature. Therefore, GA-based scavengers are best suited for products stored at temperatures higher than 10°C. At chilling temperatures, there is an disproportionate decrease in the scavenger activity in terms of the rate of O_2_ uptake. Relative humidity was shown to be the trigger for the scavenger reaction. While there was no detectable reaction below 54% RH, both the reaction rate coefficient and the scavenger capacity increased significantly at higher relative humidity in the range from 75% to 100% RH. This effect can be utilized for the application of GA-based scavengers in packaging: Packaging films containing the scavenger can be stored under typical room conditions (21°C and 54% RH) without losing their capacity. The scavenger function is only activated at higher levels of RH, i.e., when in contact with moist food products. We therefore recommend their use for products with very high water activity (*a*_*w*_>0.9) in order to ensure a fast reaction and high scavenger capacity.

## Author contributions

AP conceived and designed the experiments, did the simulations and parameter estimations and wrote the paper. JD contributed in experimental design, carried out the oxygen absorption measurement and revised the paper. MR contributed in modeling and simulation techniques and revised the paper.

### Conflict of interest statement

The authors declare that the research was conducted in the absence of any commercial or financial relationships that could be construed as a potential conflict of interest.
